# Effect of platelet-rich fibrin on cell proliferation, migration, differentiation, inflammation, and osteoclastogenesis: a systematic review of in vitro studies

**DOI:** 10.1007/s00784-019-03156-9

**Published:** 2019-12-26

**Authors:** Franz-Josef Strauss, Jila Nasirzade, Zahra Kargarpoor, Alexandra Stähli, Reinhard Gruber

**Affiliations:** 1 Department of Oral Biology, School of Dentistry, Medical University of Vienna, Sensengasse 2a, 1090 Vienna, Austria; 2 Department of Conservative Dentistry, School of Dentistry, Universidad de Chile, Av. Sergio Livingstone, 943 Santiago, Chile; 3 Department of Periodontology, School of Dental Medicine, University of Bern, Freiburgstrasse 7, 3010 Bern, Switzerland; 4 Austrian Cluster for Tissue Regeneration, Donaueschingenstrasse 13, 1200, Vienna, Austria

**Keywords:** Platelet-rich fibrin, In vitro, Growth factor, Cell proliferation, Cell migration, Cell differentiation, Anti-inflammatory agents, Osteoclastogenesis

## Abstract

**Objective:**

To systematically assess the effects of platelet-rich fibrin (PRF) on in vitro cellular behavior.

**Methods:**

A systematic electronic search using MEDLINE database was performed. In vitro studies using PRF were considered and articles published up to June 31, 2018 were screened. Eligible studies were selected based on the use of human PRF.

**Results:**

In total, 1746 titles were identified with the search terms, from these 37 met the inclusion criteria and were chosen for data extraction. In addition, 16 new studies, mainly published in 2019, were also included in the analysis resulting in 53 studies. No meta-analysis could be performed due to the heterogeneity of study designs. Included studies show that PRF enhances proliferation, migration, adhesion, and osteogenic differentiation on a variety of cell types along with cell signaling activation. Furthermore, PRF reduces inflammation, suppresses osteoclastogenesis, and increases the expression of various growth factors in mesenchymal cells.

**Summary and conclusions:**

Despite some notable differences of the studies, the overall findings suggest a positive effect of PRF on cell proliferation, migration, adhesion, differentiation, and inflammation pointing towards a therapeutic potential in regenerative dentistry.

**Clinical relevance:**

PRF serves as a reservoir of bioactive molecules to support wound healing and bone regeneration. Although the cellular mechanisms by which PRF supports the clinical outcomes remain unclear, in vitro research provides possible explanations. This systematic review aims to provide an update of the existing research on how PRF affects basic physiological processes in vitro. The overall findings suggest that PRF induces cell proliferation, migration, adhesion, and differentiation along with possessing anti-inflammatory properties further supporting its therapeutic potential in wound healing and bone regeneration.

## Introduction

Platelet-rich fibrin (PRF) is becoming an attractive and widely-used approach in regenerative dentistry. PRF is a platelet-rich plasma that undergoes natural coagulation after being separated from the red thrombus by centrifugation [1]. The evolution of PRF started with the introduction of L-PRF based on a high-speed protocol (~ 700 g for 12 min) [1]. Later on, A-PRF (~ 200 g for 8 min) and injectable PRF (~ 60 g for 3 min) with lower g-forces and centrifugation times were introduced with the overall aim to increase the number of platelets and leucocytes [2]. For this aim, the use of centrifuges with swing-out rotors has also been recommended [2]. Obviously PRF is an umbrella term that comprises various preparations and protocols, therefore a standardization of relative centrifugal forces (RCF) [3] has been suggested. Nonetheless, most of the clinical data derive from the classical L-PRF protocol [1].

**Fig. 1:**
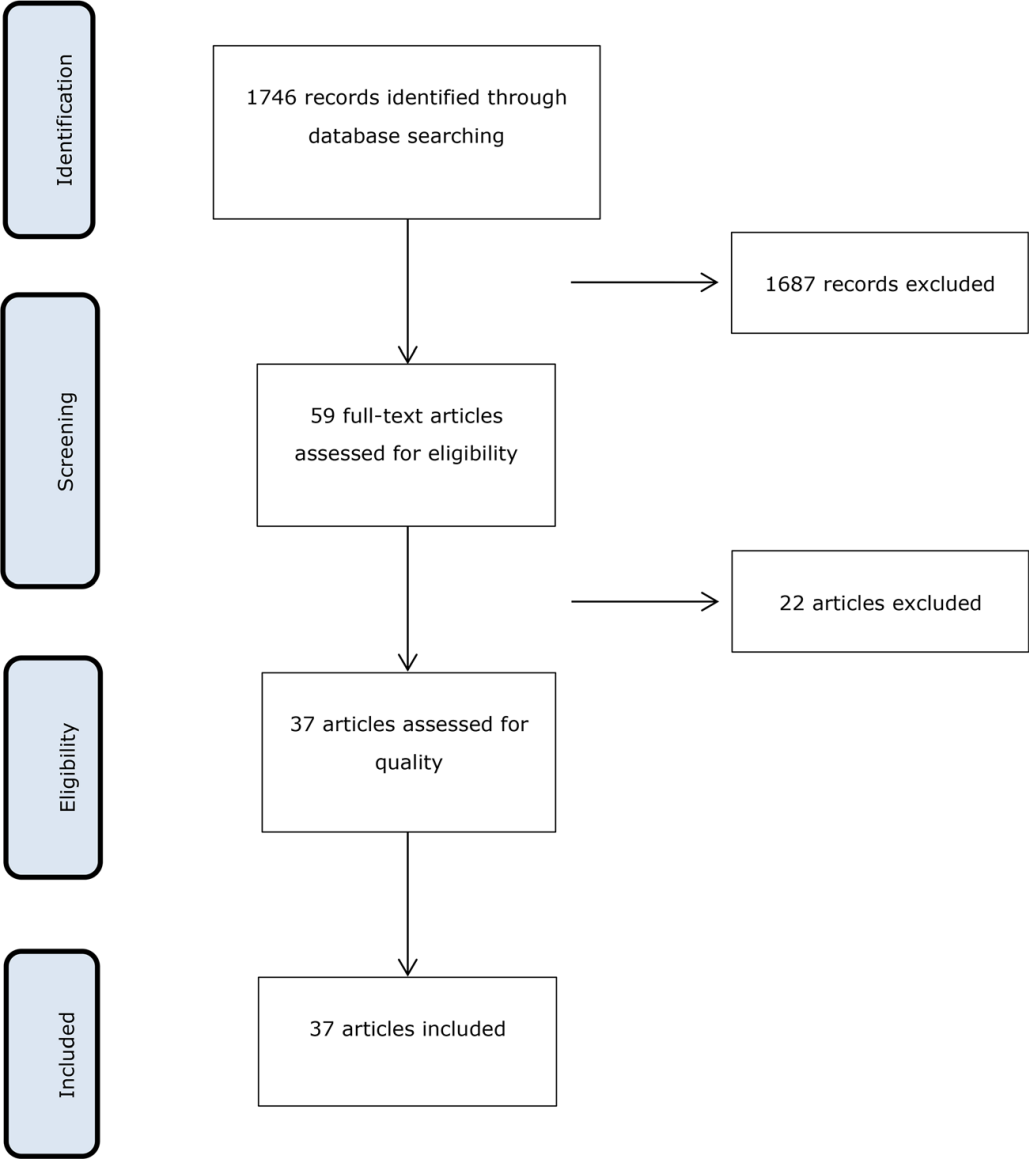
PRISMA Flow Diagram

Recent systematic reviews dealt with the clinical application of PRF in periodontal defects, periodontal plastic surgery [4], sinus floor elevation, alveolar ridge preservation, or implant therapy [5]. For example, PRF preserves the alveolar ridge after tooth extraction [6], enhances osseointegration in the early phase [7, 8] and can increase the width of keratinized mucosa around implants [9]. Even though emerging evidence indicates that local application of PRF can support the outcomes of the above-mentioned clinical indications, the underlying cellular mechanisms remain unclear. Based on the assumption that PRF supports the conserved cellular mechanisms of wound healing and bone regeneration, it can, therefore, be assumed that PRF drives the cellular responses also under in vitro conditions.

In vitro bioassays can confirm the impact of PRF on standard cellular responses such as proliferation, migration, and differentiation, all of which may predict a possible clinical efficacy. However, care should be taken when interpreting the observations, as the early hematoma that usually forms in defect sites is not represented in the in vitro assays [10]. Readers of this review should also be aware that some of the observations reported for PRF have already been shown for plasma-free leucocyte-depleted activated platelets [11–13] based on the compelling in vitro evidence gained from platelet-rich plasma [14, 15].

The cellular responses to PRF were summarized in a systematic review integrating seven in vitro studies [16]. However, given the increasing number of in vitro studies, not limited to dentistry, a revised view on today's in vitro research on PRF seems justified. This systematic review aims to provide an update of the existing research on how PRF affects basic physiological processes in vitro.

## Material and methods

### Protocol development and eligibility criteria

A protocol including all aspects of a systematic review methodology was developed prior to starting the review. This included definition of the focused question, a defined search strategy, study inclusion criteria, determination of outcome measures, screening methods, data extraction, and analysis and data synthesis.

### Defining the focused question

The following focused question was defined: “what is the effect of PRF on cell behavior in in vitro studies?”

### Search strategy

An electronic search using MEDLINE database was performed. Articles published up to June 30, 2018 were considered. No language or time restrictions were applied in the search. However, only studies written in English were included for selection.

### Search terms

The electronic search strategy included terms related to the intervention and used the following combination of key words and MeSH terms: leukocyte platelet-rich fibrin” OR “pure platelet-rich fibrin” OR “LPRF” OR “L-PRF” OR “advanced platelet-rich fibrin” OR “APRF” OR “A-PRF” OR “L-PRF Gel” OR “leukocytes“ OR “platelets” OR “blood platelets” OR “platelet” AND “in vitro techniques” OR “cytokines” OR “intercellular signaling peptides and proteins” OR “intercellular” OR intercellular signaling peptides and proteins” OR “growth factors” OR “transforming growth factor beta” OR “bone marrow” OR “stem cells” OR “macrophages” OR “osteoclasts” OR “inflammation“ OR “Cell Physiological Phenomena” OR “Cell Plasticity” OR “cell differentiation” OR “osseointegration” OR “Dental Implants.”

### Criteria for study selection and inclusion

Only in vitro studies evaluating the effect of PRF were considered.

### Exclusion criteria

In vitro studies using other kinds of platelet concentrates such as PRGF or PRP or any other platelet concentrate that required the addition of anticoagulant. Pre-clinical and in vitro studies that did not use human blood.

### Screening and selection of studies

Publication records and titles identified by the electronic search were independently screened by two reviewers (JN and ZK) based on the inclusion criteria. Discrepancies were solved by discussion among authors (RG and FJS). Cohen’s Kappa-coefficient was used as a measure of agreement between the readers. Thereafter, full texts of the selected abstracts were obtained. The two reviewers independently performed the screening process, i.e., from the MeSH term search up to the full-text examination. Then, articles that met the inclusion criteria were processed for data extraction.

### Data extraction and analysis

The inclusion criteria were applied for data extraction. The studies were classified according to study design and type of methods applied. Then, outcomes were compiled in tables. All extracted data were double-checked, and any questions that came up during the screening and the data extraction were discussed within the authors to aim for consensus.

## Results

### Selection of studies

In the original search 1746 potential references were identified in Medline which 59 were eligible after title and abstract screening (inter-reviewer agreement *κ* = 0.952). Of the 59 full-text articles, 22 did not meet the inclusion criteria and were excluded (Fig. 1) obtaining 37 studies for data extraction (Table 1). During the submission-process of the present review, 16 new studies meeting the inclusion criteria were published and therefore included for data extraction (Table 2).

**Table 1:** Included studies

Study (year)	Cell type	PRF preparation	Method	Main outcome induced by PRF
Beitzel et al. (2014) [17]	Human mesenchymal stem cells (MSCs)	3000 rpm 10 min Hardware: NR	Cell viability by live and dead staining Cell adhesion assay to the PRF-Matrix Cell proliferation by incorporation of 3H-thymidine	Cell viability maintained Increased cell adhesion No effect in cell proliferation on collagen membranes
Burnouf et al. (2012) [18]	Human embryonic kidney fibroblasts (HEK293), human osteoblastic cell line (MG-63), SIRC, NIH/3T3, periodontal ligament cells (PDL), gingival fibroblasts (GF)	700 g 12 min Hardware:^a^	Cell proliferation by an automated cell counter	Increased proliferation of all cells except for NIH/3T3 after 7 days of stimulation
Chang et al. (2010) [19]	Osteoblast cell line U2OS	3000 rpm 12 min Hardware:^a^	Cell proliferation by MTT assay Western blot for ERK phosphorylation, RANKL and OPG expression	Increased proliferation after 1, 3 and 5 days Increased ERK phosphorylation during 5 days and OPG expression during 3 days
Chang et al. (2011) [20]	Periodontal ligament fibroblasts (PDLFs)	3000 rpm 12 min Hardware:^a^	Western blot for p-ERK and OPG ALP activity	Increased ERK phosphorylation and OPG protein expression up to 5 days Enhanced ALP activity up to 5 days
Clipet et al. (2012) [21]	SaOS2 (osteoblasts), MRC5 (fibroblasts) KB (epithelial cells)	400 g 12 min Hardware: NR	Cell proliferation by SRB assay Cytotoxic assay by SRB assay Cell cycle analysis by flow cytometry tests Gene expression of cbfa1, Col1, OC and OP by RT-PCR	Increased proliferation of SaOS2, MRC5 and KB Enhanced G2M phase in SAOS2 and MRC5 lineages Up-regulation of OP and OC on SAOS2
Dereli et al. (2018) [22]	Limbal epithelial cells	PRF gel: 1000 rpm/5 min PRF: 2700 rpm/12 min Hardware:^b^	Cell viability by live and dead staining	Cell viability maintained
Dohle et al. (2018) [23]	Human outgrowth endothelial cells (OECs) primary osteoblasts (pOBs)	700 rpm 3 min Hardware:^c^	Co-culture of OECs with pOBs Angiogenic activation by immunofluorescent staining ELISA for VEGF, PDGF-BB, E-selectin and ICAM-1 Gene expression of VEGFA, ICAM1, PDGF-BB, E-selectin. BMP2 and ALP by RT-PCR	Increased VEGF concentration on pOBs at 7 days Increased of PDGF and E-Sel levels in OECs and pOBs co-cultures at the mRNA level and protein level at 24 and 72 h Increased expression of ICAM1 and ALP at 24 h in OECs and pOBs co-cultures Upregulation of VEGF expression in PRF/co-culture at 1 and 7 days Increased BMP2 expression in PRF co-cultures cultivated at 1 and 7 days
Ehrenfest et al. (2009) [24]	Osteoblasts, fibroblasts, preadipocytes, prekeratinocytes	400 g 12 min Hardware:^a^	Proliferation assay Cytotoxicity by MTT assay Osteoblast differentiation by Von Kossa and ALP staining	Increased proliferation of fibroblasts and osteoblasts at 3, 7, 14 and 21 days in a dose-dependent manner on osteoblasts Absence of cytotoxicity in all cells Increased ALP activity after 3 days and up to 28 days and increased osteoblasts differentiation after 7 days and up to 28 days
Ehrenfest et al. (2010) [25]	Human bone mesenchymal stem cells (BMSC)	400 g 12 min Hardware:^a^	Proliferation and cytotoxicity by MTT assay Osteoblastic differentiation by ALP activity and quantification of the mineralization nodules	Increased proliferation in standard and osteogenic conditions in a dose-dependent manner Absence of cytotoxicity on BMSC in standard or osteogenic conditions Increased ALP activity in a dose-dependent manner in standard and osteogenic conditions
Gassling et al. (2013) [26]	Human osteoblasts	400 g 12 min Hardware: NR	Cell viability by live and dead staining Biocompatibility and cell proliferation by lactate dehydrogenase, BrdDU, MTT and WST-1 Alkaline phosphatase activity by ELISA	Cell viability maintained Increased ALP activity
Gassling et al. (2010) [27]	Human periosteal cells	400 g 12 min 2700 rpm Hardware:^d^	Cell viability by live and dead staining Biocompatibility test by LDH test and MTT assay Cell Proliferation by BrdU	Cells seeded on PRF membranes maintained their viability PRF membranes were biocompatible No effect on proliferation
He et al. (2009)[28]	Rat calvaria osteoblasts	400 g 10 min Hardware: NR	Cell Proliferation ALP activity Mineralization assay by alizarin red staining	Increased proliferation at 5 days and enhanced mineralization at 14 days No effect on ALP activity
He et al. (2016) [29]	Human dental pulp cell (hDPC)	400 g 10 min Hardware: NR	Proliferation assay by CCK-8 Gene expression of ALP and DSPP by RT-PCR	Increased proliferation after 5 and 7 days Up-regulation of ALP and DSPP expression
Hong et al. (2018) [30]	Human Stem Cells of the Apical Papilla (SCAPs)	400 g 10 min Hardware: NR	Cell proliferation assay Cell migration assay Osteogenic differentiation by alizarin red staining Gene expression of ALP, BSP, DMP1, and DSPP by RT-PCR	Increased proliferation at 1, 3, 5 and 7 days Enhanced migration at 12 and 24 h Increased mineralization at 7 and 14 days Down-regulation of ALP, BSP, DMP1 expression but not DSPP after 7 days Up-regulation of ALP, BSP, DMP1 and DSPP expression after 14 days
Huang et al. (2010) [31]	Dental pulp cells (DPCs)	3000 rpm 10 min Hardware:^a^	Cell proliferation by MTT assay Western blot for OPG ALP activity assay	Increased DPC proliferation at 1, 3 and 5 days Up-regulation of OPG expression in a time-dependent manner Increased ALP activity
Kang et al. (2011) [32]	Human alveolar bone marrow stem cells (hABMSCs)	400 g/10 min + 230 g/10 min Hardware: NR	Cell proliferation by BrdU assay Cell migration by wound-healing assay Mineralization by alizarin red staining	Increased proliferation Reduced migration at 1 and 2 days Enhanced mineralization
Khurana et al. (2017) [33]	Dental pulp stem cells (DPSCs) periodontal ligament stem cells (PDLSCs)	3000 rpm 10 min Hardware: NR	Cell viability by trypan blue	Absence of cytotoxicity at 7 days
Kim et al. (2017) [34]	Human derived osteoblasts	400 g 10 min Hardware:^e^	Cell proliferation by MTT assay Osteoblast differentiation by Sulforhodamine B assay ALP activity	Increased proliferation at 1, 2 and 3 days Enhanced osteoblast differentiation Increased ALP activity at 2 and 3 days
Kim et al. (2017) [34]	Human dental pulp cells (HDPCs)	400 g 10 min Hardware: NR	Cell viability by MTT assay ELISA for IL-1β, IL-6, and IL-8 Western blot for VCAM1 and ICAM1 and odontoblastic differentiation markers DSP and DMP1 ALP Activity Mineralization by alizarin red staining	Absence of cytotoxicity Reduced LPS-induced pro-inflammatory cytokines IL-1b, IL-6, and IL-8 at 1 and 3 days and LPS-induced adhesion molecules VCAM1 and ICAM1 at 1 day Enhanced LPS-induced up-regulation of odontoblastic differentiation markers DSP and DMP1 at 3 days Increased ALP activity and mineralization at day 7 in LPS treated cells
Kobayashi et al. (2015) [35]	Human umbilical vein endothelial cells (HUVECs)	600 g/2 min + 500 g/4 min + 800 g/3 min Hardware:^f^	Scratch assay Western blot for VEGFR2 New blood vessel formation by CAM assay	Increased migration Enhanced phosphorylation of VEGFR2 in a dose-dependent manner Increased number of blood capillaries
Fujioka-Kobayashi et al. (2017)[36]	Human gingival fibroblasts	L-PRF: 708 g/12 min A-PRF: 200 g/14 min A-PRF+: 200 g/8 min Hardware:^c^	Cell viability by live-dead staining Cell migration assay Cell proliferation by MTS assay Gene expression of TGFβ, PDGF, and COL1a2 by RT-PCR	Cell viability maintained Increased migration Increased proliferation at 3 and 5 days Up-regulation of PDGF and COL1a2 expression and TGFβ, PDGF and COL1a2 at day 7 A-PRF+ produced the highest expression of TGFβ and COL1a2
Liang et al. (2018) [37]	Nanofat-derived stem cells (NFSCs)	2700 rpm 12 min Hardware: NR	Cell proliferation by CCK-8 Gene expression of VEGF, bFGF, PDGF and TGFβ by RT-PCR Protein levels by Western blot	Increased proliferation Increased expression and protein levels of VEGF, bFGF, PDGF and TGFβ Enhanced osteogenic, adipogenic and chondrogenic differentiation
Miron et al. (2017) [38]	Human gingival fibroblasts (HGF)	I-PRF:700 rpm 60 g 3 min Hardware:^c^	Cell viability by live and dead assay Cell migration by Boyden chamber Cell proliferation by MTS assay Gene expression of TGFβ, PDGF and COL1a2 by RT-PCR	Absence of cytotoxicity Increased cell migration Increased proliferation after 5 days Upregulation of COL1a2 and PDGF expression after 3 days, and TGFβ and COL1a2 after 7 days
Moradian et al. (2017) [39]	Human bone marrow mesenchymal stem cells (BMMSCs)	400 g 10 min Hardware: NR	Cell proliferation by MTT assay	Increased proliferation at 7 days
Schär et al. (2015) [40]	Human bone marrow-derived MSC and HUVEC	400 g 12 min Hardware:^b^	Cell migration assay by Boyden chambers	Increased migration of MSC and HUVEC cells at 7 days and at 8 h, respectively
Park et al. (2018) [41]	Human umbilical vein endothelial cell (HUVEC)	400 g 12 min Hardware:^a^	Cell proliferation by MTT assay Cytotoxicity assay by adenylate kinase (AK) release from dead cells Cell migration by Boyden chamber assay Cell attachment by Green Nucleic Stain-Kit	Increased proliferation, migration and attachment of cells by coating porcine-matrices with PRF Increased cytotoxicity at day 1
Passaretti et al. (2014) [42]	HUVEC, skin fibroblasts	400 g 12 min Hardware:^a^	Cell proliferation by Bürker chamber counting and automated cell counter	Increased cell proliferation at 24 h
Saeed et al. (2017) [43]	Dental pulp stem cells (DPSCs)	2700 rpm 12 min + 1800 rpm/5 min Hardware: NR	Cell proliferation by MTS assay Osteogenic differentiation by alizarin red assay	Reduced cell viability at 1 and 3 days by most PRF concentrations Enhanced osteogenic differentiation at 7 days
Vahabi et al. (2015) [44]	Human gingival fibroblasts cell line (HGF)	400 g 12 min Hardware: NR	Viability and proliferation by MTT assay	Cell viability maintained during the first 24 h, at 48 and 72 h cell viability is reduced Increased proliferation only the first 24 h
Wang et al. (2018) [45]	Human primary osteoblasts	I-PRF:700 rpm 60 g 3 min Hardware:^c^	Cell viability by live and dead staining Cell migration by polyethylene terephthalate cell culture inserts Cell adhesion assay by staining cells with 4′,6-diamidino-2-phenylindole Cell proliferation by CCK-8 ALP activity assay Mineralization by alizarin red staining Gene expression of COL1A, Runx2, ALP, OCN by RT-PCR Immunofluorescence staining for OC expression	Cell viability maintained Increased migration at 1 day No effect on cell adhesion Increased proliferation at 3 and 5 days Enhanced ALP activity and mineralized nodule formation Up-regulation of ALP expression at 3 days and OCN, Runx2 and COL1A at 14 days Increased staining intensity of OCN
Wang et al. (2017) [46]	Human gingival fibroblasts	700 rpm 60 g 3 min Hardware:^c^	Cell viability by live-dead staining assay Cell migration assay by polyethylene terephthalate cell culture inserts Cell adhesion assay Cell proliferation by CCK-8 Gene expression of PDGF, TGFβ and COL1a1 and FN1 Collagen Type I Staining Immunostaining	Cell viability maintained irrespective of the titanium surface Increased migration in tissue culture plates and titanium surfaces No effect on cell adhesion Increased cell proliferation after 3 and 5 days Upregulation of PDGF, TGF-b, COL1 and FN1 expression levels on all surfaces Increased fluorescence intensity of collagen type 1 on all surfaces.
Wei et al. (2017) [47]	Nanofat-derived Stem Cells (NFSCs)	2700 rpm 12 min Hardware: NR	Proliferation assay by CCK-8 Gene expression of adipogenic differentiation markers PPARγ2, C/EBPα, and ADD1 by RT-PCR	Increased proliferation after 3 days Enhanced adipogenic differentiation at 14 days in a dose dependent-manner Up-regulation of PPARγ2, C/EBPα, and ADD1 expression
Wirohadidjojo et al. (2016)[48]	Ultraviolet-A (UVA)-irradiated human dermal fibroblasts (HDFs)	400 g 12 min Hardware: NR	Proliferation assay by MTT assay Collagen deposition assay Cell migration rate assay	Increased proliferation, migration and collagen deposition in UVA-irradiated HDFs
Woo et al. (2016) [49]	Human dental pulp cells (HDPCs)	400 g 10 min Hardware: NR	Cell viability by MTT and MTA assay ALP activity assay ALP staining Alizarin red staining for mineralization formation Western Blot for DSP,DMP1, BMP 2/4, phospho-smad1/5/8	Cell viability maintained at 48 h Increased protein levels of DSP and DMP1 and enhanced ALP activity. Increased mineralization Activation of BMP 2/4 signaling and phosphorylation of SMAD1/5/8
Wu et al. (2012) [50]	Human osteoblast cell line (U2OS)	3000 rpm 12 min Hardware:^c^	Cell attachment assay by WST-1 assay Cell proliferation assay Western blot analysis for p-Akt, HSP 47 and LOX	Increased cell attachment the first 3 h Increased proliferation at 1, 3 and 5 days. Enhanced Akt phosphorylation, HSP 47 expression, and LOX expression in U2OS cells
Xu et al. (2016) [51]	Human breast adipose-derived stem cells (HBASCs)	2700 rpm 12 min Hardware: NR	Attachment of HBASCs on scaffolds in the presence of PRF, Rg1 or both by fluorescence imaging	Increased proliferation and attachment on scaffolds
Zhao et al. (2013) [52]	Periodontal ligament stem cells (PDLSCs)	400 g 10 min Hardware: NR	Cell viability and proliferation assay by MTT assay ALP activity Gene expression of BSP, OCN, ColI, and CP23 by RT-PCR	Cell viability maintained Increased proliferation during 7 days Reduced ALP activity at 7 days Down-regulation of BSP and OCN expression at 7, 14 and 21 days Up-regulation PDL-related genes ColI and CP23

*Note. NR* not reported, *MTT* 3-(4,5-Dimethylthiazol-2-Yl)-2,5-Diphenyltetrazolium Bromide, *ERK* extracellular signal-regulated kinase, *RANKL* receptor activator of NF-β ligand, *OPG* osteoprotegerin, *ALP* alkaline phosphatase, *SRB* sulforhodamine B, *cbfa1* core-binding factor subunit alpha-1, *LPS* lipopolysaccharide, *VEGF* vascular endothelial growth factor, *ICAM-1* intercellular adhesion molecule 1, *ELISA* enzyme-linked immunosorbent assay, *BMP* bone morphogenetic protein, *RT-PCR* reverse transcription polymerase chain reaction, *BrdDU* bromodeoxyuridine, *WST-1* water soluble tetrazolium-1, *LDH* lactate dehydrogenase, *CCK-8* cell counting kit-8, *BSP* bone sialoprotein, *DMP* dentin matrix protein, *MTS* 3-(4,5-dimethylthiazol-2-yl)-5-(3-carboxymethoxyphenyl)-2-(4-sulfophenyl)-2H-tetrazolium), *TGFβ* transforming growth factor-β, *COL1a2* collagen type I alpha 2, *bFGF* basic fibroblast growth factor, *Runx2* runt-related transcription factor 2, *OCN* osteocalcin, *FN1* fibronectin, *ECM* extracellular matrix, *PPARγ2* peroxisome proliferator-activated receptor, *C/EBPα* CCAAT-enhancer-binding proteins

^a^PC- 02, Nice, France

^b^Hettich EBA20, Tuttlingen, Germany

^c^Duo Centrifuge, Nice, France

^d^Eppendorf Centrifuge 5702, Hamburg, Germany

^e^Gyrozen 406, Daejeon, Korea

^f^Medifuge centrifugation system, Santa Sofia, Italy

**Table 2:** Included studies

Study (year)	Cell type	PRF preparation	Method	Main outcome induced by PRF
Bucur et al. (2019) [53]	Fibroblast cell line L929	200 g 14 min Hardware: NR	Cell proliferation and migration using RCTA Scratch assay	No effect on proliferation neither on migration
Elgamal et al. (2019) [47].	Adipose mesenchymal stem cells (MSC)	1500 rpm 14 min Hardware: NR	Cell proliferation by MTT assay	Increased proliferation
Gervois et al. (2019) [54]	Human dental pulp stem cells (hDPSCs), neural stem cell (NSC)	400 g 12 min Hardware:^c^	Metabolic activity assay by MTT assay Cell proliferation by PI assay Cell migration by transwell migration assay	Decreased and increased metabolic activity, dependent on the PRF concentration Increased proliferation of hDPSCs but no effect on NSC Increased migration of NSC
Gomez et al. (2019) [55]	Endothelial cells (EC)	2000 rpm 7 min Hardware: NR	Counting of the cells viewed under phase-contrast microscope	Increased cell growth
Herrera-Vizcaíno et al. (2019) [56]	Human dermal vascular endothelial cells (HDMECs) Human fibroblasts (HF)	44 g or 710 g 8 min Hardware:^a^	Immunostaining of endothelial cells marker CD31 Immunohistochemical detection of CD31 Protein levels of VEGF, PDGF-BB and TGFβ by ELISA	Increased CD31-positive cells Increased concentration of PDGF-BB of TGFβ at day 4 in HDMECs and HF No effect on VEGF
Kargarpour et al. (2019) [57]	Murine primary macrophages, RAW264.7 cells	1570 rpm 12 min Hardware:^d^	Cell viability by MTT assay and live-dead staining Cell proliferation by BrdU Caspase 3 activity assay Western blot for cleaved caspase 3 Gene expression of osteoclast marker genes TRAP, Cathepsin K, DCSTAMP, NFATc1, OSCAR and pro and anti-apoptotic marker genes, caspase-3, Bax and Bcl2L1 by RT-PCR TRAP staining Pit formation assay	Maintenance of cell viability and enhanced cell proliferation Reduced caspase 3 activity and suppression of cleaved caspase-3 Supression of osteoclastogenesis Suppression of the expression of TRAP, Cathepsin K, DCSTAMP, NFATc1, OSCAR Reduced number of multinucleated TRAP positive cells Reduced pit formation on dentine slices
Kasnak et al. (2019) [58]	Human oral keratinocyte (HMK) cells	2700 rpm 15 min Hardware:^e^	Cell proliferation by CellTiter 96 assay Protein levels of IL1β, IL1Ra, IL8, MCP1, and VEGF by ELISA	Increased proliferation on titanium and hydroxyapatite discs Increased concentration of IL1β, IL1Ra, IL8, MCP1, and VEGF on titanium and hydroxyapatite discs
Li et al. (2018) [59]	Human periodontal ligament cells (hPDLCs)	750 g 12 min + 500 g 5 min Hardware: NR	Cell proliferation using CCK-8 ALP activity assay Mineralization assay by alizarin red staining Gene expression of RUNX2, Osterix and Osteocalcin by RT-PCR Protein expression of RUNX2 by Western blot	Increased proliferation at day 1, 2 and 3 Increased ALP activity at 7 and 14 days Increased mineralization at 14 days Increased expression of RUNX2, Osterix at 5 and 7 days and Osteocalcin at 7 days Increased protein expression of RUNX2 at day 5
Mahendran et al. (2019)[60]	Fibroblast cell line L929	3000 rpm 10 min Hardware: NR	Cell viability by MTT assay	Maintenance of cell viability
Mudalal et al. (2019) [61]	Human gingival fibroblast	3000 rpm 12 min Hardware:^b^	Gene expression of IL1β, IL6 and TNFα by RT-PCR	Decreased expression of IL1β, IL6 and TNFα
Nasirzade et al. (2019) [62]	Murine primary macrophages, Raw 264.7 cells	1570 rpm 12 min Hardware:^d^	Gene expression of M1 marker genes IL1β, IL6; M2 genes Arg1, Ym1 and lipoxygenases, ALOX12, and ALOX15 by RT-PCR IL6 levels by ELISA NF-휅B intracellular translocation by Immunofluorescent	Decreased expression of IL1β and IL6 Increased expression of Arg1, Ym1 and lipoxygenases Increased IL6 protein level Reduced intracellular translocation of NF-휅B
Ratajczak et al. (2018) [63]	HUVEC	400 g 12 min Hardware:^c^	Cell proliferation by MTT assay and propidium iodide assay Cell migration by transwell migration assay Angiogenic potential by Tube Formation assay	Increased proliferation Enhanced migration Increased tube formation
Steller et al. (2019) [64]	Primary human osteoblasts (OB)	400 g 10 min Hardware:^f^	Viability by MTT assay Cell adhesion to titanium surface by RTCA and cell wash assay	Maintenance of cell viability No effect on adhesion to titanium surface
Steller et al. (2019) [64]	Human gingival fibroblasts (GF), human osteoblasts (hOB)	400 g 10 min Hardware:^f^	Cell viability by MTT assay Cell migration by scratch assay	Cell viability maintained at 24 and 72 h in OB and at 72 h in GF Increased migration of GF and OB at 24, 48 and 72 h Increased proliferation
Verboket et al. (2019)[40]	Bone marrow monuclear cells (BMC)	60 g 3 min 208 g 8 min Hardware:^a^	Metabolic activity by MTS assay Gene expression of VEGFA, ICAM1, MMP2, MMP7, MMP9, TGF-β1, BCL2, BAX, ALP, COL1a1, FGF23, and OPN by RT-PCR Determination of apoptosis using Annexin-V-staining	Increased metabolic activity at day 14 Increased expression of SPPI at day 2 and 7, TGFβ, MMP2, at day 7 and MMP9 at day 14 No effect on ICAM, ALP, COL1a1, FGF23 and other genes. PRF did not induce apoptosis
Wang et al. (2019) [65]	Dermal skin fibroblast cell	60 g 3 min Hardware: NR	Cell viability using live and dead staining Cell migration assay Cell proliferation using CCK-8 Gene expression of PDGF, TGFβ, COL1a1, and FN1 by RT-PCR Immunofluorescent staining of collagen type I	Maintenance of cell viability Increased the migration Increased proliferation at day 3 and 5 Increased expression of PDGF, TGFβ, COL1a1, and FN1 at 3 and 7 days. Increased collagen type I staining

*Note. NR* not reported, *MTT* 3-(4,5-Dimethylthiazol-2-Yl)-2,5-Diphenyltetrazolium Bromide, *ALP* alkaline phosphatase, *COL1A1* collagen 1 alpha 1, *RT-PCR* reverse transcription polymerase chain reaction, *ELISA* enzyme-linked immunosorbent assay, *VEGF* vascular endothelial growth factor, *ICAM1* intercellular adhesion molecule, *SPPI* osteopontin, *PDGF-BB* platelet-derived growth factor, *PI* propodeum iodide, *BDNF* brain-derived neurotrophic factor, *CCK-8* cell counting kit-8, *TGFβ* transforming growth factor-β, *TRAP* tartrate-resistant acid phosphatase, *DCSTAMP* dendritic cell-specific transmembrane protein, *NFATc1* nuclear factor of activated T-cells, *OSCAR* osteoclast-associated receptor, *Bax* Bcl2-associated x protein, *Bcl2* B cell lymphoma 2, *MCP-1* monocyte chemotactic protein-1, *MTS* 3-(4,5-dimethylthiazol-2-yl)-5-(3-carboxymethoxyphenyl)-2-(4-sulfophenyl)-2H-tetrazolium), *FGF23* basic fibroblast growth factor, *TNF-α* tumor necrosis factor, *Arg1* arginase-1, *ALOX* arachidonate lypoxigenase, *NF* nuclear factor kappa-light-chain-enhancer of activated B cells, *RTCA* real-time-cell analyzer assay, *M-CSF* macrophage colony stimulating factor, *MMP* matrix metalloproteinase, *FN1* fibronectin

^a^Duo Centrifuge, Nice, France

^b^Eppendorf Centrifuge 5702, Hamburg, Germany

^c^Intraspin TM, Intra-Lock International, Boca Raton, FL

^d^Z 306 Hermle Universal Centrifuge, Wehingen, Germany

^e^SL8R, Thermo Fisher Scientific, Waltham, MA

^f^Allegra X-12R-Centrifuge, Brea, California

### Proliferation

PRF increased proliferation of mesenchymal cells, for example from bone of different origin [19, 24–26, 28, 45, 50, 66, ], bone marrow [32, 39], periosteum [27], adipose tissue [37, 47, 68], and skin [65, 48]. Also, fibroblasts from gingiva [38, 44], periodontal ligament [18, 52, 59], papilla [30], and dental pulp responded to PRF with increased proliferation [29, 31, 43, 54]. These observations were reproduced in embryonic kidney fibroblasts and in various cell lines such as HEK293, MG-63 osteosarcoma cells, human oral keratinocytes, SIRC, and 3T3 cells [18]. Mesenchymal cells, endothelial cells [23, 42, 55, 63], epithelial cells [22], and macrophages [69] also responded to PRF with increasing proliferation. In contrast, PRF failed to induce proliferation of L929 fibroblasts [53] and human mesenchymal stem cells on collagen scaffolds [17]. In general, PRF maintained cell viability [33, 63–66, ] without inducing apoptosis [40]. Overall, there is a general consensus that PRF has a potent mitogenic activity.

### Migration

There are various methods to identify the impact of PRF on cell migration including the scratch assay [70] and the traditional Boyden chamber approach [71]. Regardless of the method used, PRF increased the migration of neural stem cells [54] along with cells of the mesenchymal lineage isolated from bone [45, 64], bone marrow [72], gingiva [38, 64, 36], apical papilla [30], and skin [65, 48]. Similarly, endothelial cells responded to PRF with an increased migration [63, 72, 41]. In contrast, an inhibitory effect of PRF on cell migration was also observed on bone marrow cells but likely due to the aggregation and proliferation effect of PRF that precedes migration [32]. Likewise, in one recent study, PRF failed to induce migration on L929 fibroblasts [53]. However, the general view is that PRF supports cell motility.

### Alkaline phosphatase and alizarin red staining

The main early marker of osteogenic differentiation is alkaline phosphatase [73]. Various studies showed that PRF increases the expression or the activity of alkaline phosphatase in cells of the mesenchymal lineage isolated from bone [45, ], bone marrow [25], apical papilla [30], dental pulp [31, 34, 43, 49], periodontal ligament [59, 74], osteosarcoma cell lines [21], and other tissues [24]. Moreover, PRF increased mineralized nodules in cells from dental pulp [34, 43, 49], calvaria bone [28], bone marrow [32], and periodontal ligament [59]. Conversely, one study showed an inhibitory effect of PRF on alkaline phosphatase activity [52]. In two other reports, PRF failed to change alkaline phosphatase activity and did not change alkaline phosphatase expression in rat calvaria osteoblasts [28] and bone marrow cells [40], respectively. Taken together, all but three studies reported an increase of alkaline phosphatase in response to PRF exposure.

### Growth factors and extracellular matrix

PRF caused a moderate expression of various growth factors in mesenchymal and endothelial cells such as TGFβ [38, 46, 52, 56, 65, 36], PDGF [23, 38, 40, 46, 52, 56, 65, 36], and VEGF [23, 37, 58]. Dental pulp cells treated with PRF increased expression of dentin sialoprotein and dentin matrix protein 1 [29, 34, 49]. With respect to changes in the expression of extracellular matrix protein, PRF increased collagen type 1 in mesenchymal cells of the bone [45], skin [65], and gingiva [38, 73]. Likewise, PRF increased the expression of osteopontin, MMP2, and MMP9 in human bone marrow cells [40]. Conversely, PRF reduced the expression of bone sialoprotein and osteocalcin along with a transient downregulation of collagen type 1 in periodontal ligament cells [52]. Similarly, a downregulation of bone sialoprotein, dentin matrix protein 1, and dentin sialoprotein in cells from the papilla was reported [30]. It should be noted, however, that this downregulation disappeared after 14 days of stimulation [30]. In general, the reported increase of gene expression by PRF is moderate.

### Cell adhesion

Cell adhesion proteins were enhanced by PRF, for example, ICAM-1 and E-selectin in cocultures of osteogenic and endothelial cells [23] and ICAM-1 in pulp cells [34]. Furthermore, PRF supported adhesion of mesenchymal cells [17], HUVEC [41], U2OS [50], and HBASC [51] on different scaffolds. These positive results nonetheless were not replicated on titanium surfaces [46] and culture plates [45]. Together, these observations suggest that in the majority of experiments, PRF could support cell adhesion.

### Cell signaling, inflammation, and osteoclastogenesis

PRF enhanced the phosphorylation of Akt, heat shock protein 47 and lysis oxidase in osteosarcoma cells [50], and VEGFR2 in endothelial cells [35]. PRF enhanced phosphorylation of ERK in osteosarcoma cells [19], and periodontal fibroblasts [74] along with an increase in OPG expression in both cell types. This PRF-induced OPG expression was also reported on dental pulp cells [31]. Moreover, PRF reduced LPS-induced cytokine production in pulp cells and enhanced the up-regulation of odontoblastic differentiation markers DSP and DMP-1 in these cells [34]. Similarly, PRF suppressed the LPS- and saliva-induced pro-inflammatory cytokines on primary and RAW264.7 macrophages and attenuated the translocation of NF-κB into the nucleus [69]. This anti-inflammatory effect was replicated in gingival fibroblasts [61]. In addition, in dental pulp cells, PRF increased DSP and DMP1 expression along with an activation of BMP 2/4 signaling and phosphorylation of SMAD1/5/8 cascade [49]. Osteoclasts originate from hematopoietic progenitors and in the presence of the survival factor (M-CSF) and RANKL differentiate into osteoclasts staining positive for TRAP. PRF suppressed the expression of osteoclast marker genes TRAP, DCSTAMP, NFATc, and OSCAR. Altogether, these results suggest that PRF can affect central signaling pathways, possesses an anti-inflammatory effect, and is capable of inhibiting osteoclastogenesis [57].

## Discussion

This systematic review encompassed in vitro studies using PRF and can be viewed as an extension of the previous work of Miron et al. [16]. Our aim was to gather the current in vitro evidence on cellular responses to PRF. Despite the steadily increasing number of in vitro studies, much of the available evidence has focused on confirming similar findings. The majority of studies assessed the impact of PRF on proliferation, adhesion, migration, and differentiation mainly on mesenchymal cells and to some extent, endothelial and epithelial cells. Overall, PRF triggered an increase in the above-mentioned parameters and revealed anti-inflammatory properties. PRF also showed a moderate but consistent capacity to modulate the expression of target genes activating different signaling pathways.

A meta-analysis could not be performed as the included studies revealed heterogeneity in terms of study design, evaluation methods, outcome measures, and observation periods. Besides the original L-PRF protocol, other PRF protocols were used, however, most studies did not provide enough details. These details are of importance as with different protocols [3], i.e., centrifugation time and g-force, characteristics such as the release of growth factors or the content of living cells are substantially changed [75]. For instance, by reducing the g-force, there is an improvement in growth release and cell content. This finding is considered one of the major innovations in PRF leading to the development of new protocols including advanced platelet-rich fibrin (A-PRF+), injectable PRF (i-PRF), and liquid PRF (fluid-PRF). In addition, there are other factors that were not considered in the different preparation protocols such as the centrifugation tubes which have a strong impact on the clot size [76]. Indeed, the silica used to coat plastic tubes might contaminate PRF and thereby provoking inflammation [77]. Likewise, differences in g-forces, blood volume, hematocrit levels, centrifugation time, and handling of PRF membranes impede an accurate comparison between the protocols. Furthermore, PRF lysates, PRF conditioned medium, and PRF exudates should also be distinguished from traditional protocols. Although these issues are at the heart of scientific discussion [78, 79], the main in vitro findings are rather consistent.

Successful tissue regeneration and osseointegration rely on the response of the surrounding cells. These biological processes inevitably require proliferation, migration, and differentiation of cells at the treatment site.

PRF consistently increased cell proliferation irrespective of the cell type and PRF preparation. One interesting setting was the increased cell proliferation on collagen matrices [72] and titanium surfaces [46] upon PRF coating. It is worth noting that two studies found a conspicuously reduced proliferation in gingival fibroblast [44] and dental pulp stem cells [43]. It is difficult, however, to determine why PRF led to a decline in cell proliferation. PRF membranes covering cells might decrease oxygenation [44]. Nonetheless, PRF preparation without providing enough details complicates the interpretation of the data [43]. Despite these shortcomings, the majority of the in vitro studies suggest a mitogenic activity of PRF for various cell types that might be attributed to the strong mitogen PDGF released by activated platelets [13, 80].

Cell migration was positively induced in all but two of the selected studies. This chemotactic effect is likely due to the presence of growth factors contained in platelets such as PDGF [12]. This growth factor, for example, pushes proliferation of osteogenic cells in vitro [81]. In addition, endothelial cells followed a similar pattern of displaying an increase in migration upon exposure to PRF [40]. Although the activation of platelets might account for these observations [11], the precise mechanism remains to be elucidated. In support of the mitogenic and chemotactic activity, PRF enhanced the phosphorylation of Akt [50], and ERK [19, 74] similar to what is observed in isolated platelets [12]. Conversely, inhibition of migration by PRF has been reported in alveolar bone marrow cells [32]. This effect might be explained by the aggregation and proliferation effect of PRF that precedes migration and also by methodological differences, which precludes an interpretation and a comparison with the other studies [3]. Despite these inconsistencies, PRF is able to induce cell migration, likely due to the presence of growth factors such as PDGF with chemotactic activity.

Cell differentiation is commonly assessed by means of measuring alkaline phosphatase and alizarin red staining. Various studies showed that PRF increases the expression or the activity of alkaline phosphatase in cells of the mesenchymal lineage [24, 25, 29–31, 34, 43, 45, 49, 65, 59, 74]. Some data, nonetheless, are conflicting since PRF can also reduce alkaline phosphatase activity [52] consistent with the effects of supernatants of isolated platelets [12]. This reduction may be attributed to TGF-β [82] and PDGF [12]. On the other hand, the increased mineralized nodules elicited by PRF in cells from dental pulp [34, 43, 49], calvaria bone [28], bone marrow [32], and periodontal ligament [59] appear to be a consequence of the enhanced proliferation, alkaline phosphatase activity, and production of collagen matrix. These in vitro findings, however, have to be interpreted with caution as proliferation and differentiation do not occur simultaneously [73].

With respect to growth factors such as TGFβ, PDGF, and VEGF, PRF moderately increased their expression. Regarding extracellular matrix proteins, PRF moderately increased the expression of collagen type 1, which is a known TGFβ target gene [83, 84] in mesenchymal cells of various origins. In line with collagen type 1 synthesis, PRF activates the expression of HSP47 and lysine oxidase [50]. Cell adhesion proteins were enhanced by PRF [23], however, they are not necessarily responsible for the increased cell adhesion on different scaffolds [51]. Together, these observations suggest that PRF induces moderate changes in gene expression. In contrast, recent data at our lab indicate a robust activation of TGFβ target genes IL11, PRG4, and NOX4 by PRF lysates (Di Summa et al. unpublished observation). TGF-β couples osteogenesis with angiogenesis by providing a pro-osteogenic microenvironment in vivo [62]. As TGF-β induces pro-osteogenic factors and TGF-β type 1 receptor inhibitor rescues uncoupled bone remodeling in vivo [62], PRF-derived TGF-β may support bone regeneration.

This systematic review revealed an anti-inflammatory effect of PRF. Moreover, during the submission process of the present review, new studies were published highlighting these anti-inflammatory effects of PRF. For example, PRF reduced the LPS-induced proinflammatory cytokine release in gingival fibroblasts [61]. In addition, we have recently shown that PRF reduces the expression of the M1 marker genes interleukin 1β (IL1β) and interleukin 6 (IL6) in bone marrow macrophages [69]. This anti-inflammatory effect might be explained by the high amounts of TGFβ in PRF [73] capable of modulating the M1 and M2 polarization along with the generation of pro-resolving lipid mediators [69]. Additionally, PRF induces the expression of the M2 markers arginase-1 and chitinase-like 3 (Chil3 or YM1) thereby assisting a M1-to-M2 transition [69]. Since dental implants activate the immune system during the early stages of osseointegration [85], the addition of PRF may support a M2 polarization reducing the time lag for osseointegration and bone regeneration. Notably, PRF can also decrease the formation of osteoclast-like cell in a murine bone marrow culture [57]. Similar findings were also reported in peripheral blood mononuclear cells derived CD14^+^ cells [86]. These observations are of particular interest since the favorable effects of PRF in alveolar ridge preservation [6] might be partly explained by an inhibition of osteoclastogenesis. Thus, accumulating evidence suggest that PRF possesses an anti-inflammatory activity and is capable of suppressing osteoclastogenesis.

PRF is a potent inducer of the in vitro angiogenic process indicated by endothelial proliferation, migration, and tube formation. PRF supports microvessel-like structures [23, 56] and induces blood vessel formation in the chorioallantoic membrane assay [63]. Apart from in vitro angiogenesis, a recent report described an antimicrobial effect of PRF. In that study, both PRF membranes and PRF exudates had an antimicrobial effect against *P. gingivalis*, a key periodontal pathogen [87]. Those findings support the rationale of using PRF as an adjunctive therapy for peri-implantitis [88]. These observations are also in line with previous data on purified activated platelet showing an angiogenic [11] and antimicrobial effect [89]. Overall, these findings imply that PRF possesses angiogenic and antimicrobial properties.

We recognize that the present report has a number of limitations. PRF is widely used in regenerative dentistry, however, in vitro models represent only a narrow aspect of wound healing and bone regeneration neglecting the holistic nature of an in vivo model. Furthermore, and considering that wound healing and bone regeneration involve granulocytes, lymphocytes and other cell types, today’s PRF research only covers a restricted spectrum of cells. It should also be noted that the same stimuli may play different roles depending on the differentiation stage of the target cell. For example, our group demonstrated that PRF membranes inhibit the formation of osteoclasts in bone marrow cultures [57]. This inhibition, however, did not occur when osteoclastogenesis had already started [57].

Future studies should, for example, include research on the immigration and activation of granulocytes and how PRF might control the resolution of inflammation. Moreover, and considering the importance of centrifugation tubes and the possible impact of silica coating, more studies investigating this issue are needed for the optimization of PRF. Finally, the overall question of whether the in vitro PRF research reflects the clinical reality serving as a surrogate parameter to adapt the current PRF protocols remains to be clarified.

## Conclusion

Despite some notable differences of the included studies, the overall findings suggest a benefit of PRF on cell proliferation, migration, adhesion, differentiation, and inflammation pointing towards a therapeutic potential in wound healing and regeneration.
